# Atomic layer deposition coating of carbon nanotubes with zinc oxide causes acute phase immune responses in human monocytes *in vitro* and in mice after pulmonary exposure

**DOI:** 10.1186/s12989-016-0141-9

**Published:** 2016-06-08

**Authors:** Erinn C. Dandley, Alexia J. Taylor, Katherine S. Duke, Mark D. Ihrie, Kelly A. Shipkowski, Gregory N. Parsons, James C. Bonner

**Affiliations:** 1 Department of Chemical & Biomolecular Engineering, North Carolina State University, Raleigh, North Carolina 27695 USA; 2 Toxicology Program, Department of Biological Sciences, North Carolina State University, Campus Box 7633, Raleigh, North Carolina 27695-7633 USA

**Keywords:** Atomic layer deposition, Carbon nanotubes, Pulmonary fibrosis, Inflammation

## Abstract

**Background:**

Atomic layer deposition (ALD) is a method for applying conformal nanoscale coatings on three-dimensional structures. We hypothesized that surface functionalization of multi-walled carbon nanotubes (MWCNTs) with polycrystalline ZnO by ALD would alter pro-inflammatory cytokine expression by human monocytes in vitro and modulate the lung and systemic immune response following oropharyngeal aspiration in mice.

**Methods:**

Pristine (U-MWCNTs) were coated with alternating doses of diethyl zinc and water over increasing ALD cycles (10 to 100 ALD cycles) to yield conformal ZnO-coated MWCNTs (Z-MWCNTs). Human THP-1 monocytic cells were exposed to U-MWCNTs or Z-MWCNTs in vitro and cytokine mRNAs measured by Taqman real-time RT-PCR. Male C57BL6 mice were exposed to U- or Z-MWCNTs by oropharyngeal aspiration (OPA) and lung inflammation evaluated at one day post-exposure by histopathology, cytokine expression and differential counting of cells in bronchoalveolar lavage fluid (BALF) cells. Lung fibrosis was evaluated at 28 days. Cytokine mRNAs (IL-6, IL-1β, CXCL10, TNF-α) in lung, heart, spleen, and liver were quantified at one and 28 days. DNA synthesis in lung tissue was measured by bromodeoxyuridine (BrdU) uptake.

**Results:**

ALD resulted in a conformal coating of MWCNTs with ZnO that increased proportionally to the number of coating cycles. Z-MWCNTs released Zn^+2^ ions in media and increased IL-6, IL-1β, CXCL10, and TNF-α mRNAs in THP-1 cells in vitro. Mice exposed to Z-MWCNTs by OPA had exaggerated lung inflammation and a 3-fold increase in monocytes and neutrophils in BALF compared to U-MWCNTs. Z-MWCNTs, but not U-MWCNTs, induced IL-6 and CXCL10 mRNA and protein in the lungs of mice and increased IL-6 mRNA in heart and liver. U-MWCNTs but not Z-MWCNTs stimulated airway epithelial DNA synthesis in vivo. Lung fibrosis at 28 days was not significantly different between mice treated with U-MWCNT or Z-MWCNT.

**Conclusions:**

Pulmonary exposure to ZnO-coated MWCNTs produces a systemic acute phase response that involves the release of Zn^+2^, lung epithelial growth arrest, and increased IL-6. ALD functionalization with ZnO generates MWCNTs that possess increased risk for human exposure.

**Electronic supplementary material:**

The online version of this article (doi:10.1186/s12989-016-0141-9) contains supplementary material, which is available to authorized users.

## Background

The use of carbon nanotubes (CNTs) in industrial and academic settings has increased dramatically in the last decade. CNTs are used in many different areas including electronics, energy storage, sensors, conductive coatings, capacitors, filtration, and drug delivery [1, 2]. Despite these many potential applications, CNTs share geometric similarities with asbestos and thus there is concern for pulmonary fibrosis, a fatal disease characterized by progressive scar tissue accumulation in the lungs [3]. Rodent studies demonstrate that multi-walled CNTs (MWCNTs) or single-walled CNTs (SWCNTs) delivered to the lungs of rats and mice by inhalation, oropharyngeal aspiration or intratracheal instillation cause fibrosis, suggesting a similar health risks to humans [1, 4–6]. Moreover, MWCNTs or SWCNTs activate pro-fibrotic signaling pathways and stimulate the production of soluble pro-fibrotic mediators by cultured lung cells, including fibroblasts and monocytes/macrophages suggesting that these in vitro cell models are valuable for predicting the inflammatory and fibrotic potential of CNTs in vivo [5–9].

Atomic layer deposition (ALD) is a thin-film deposition technique that utilizes self-limiting surface reactions to achieve conformal thin film coatings with precise sub–nanometer thickness control on complex 3D surfaces, including MWCNTs [10–12]. ALD allows for thin-film surface modification of MWCNTs with a variety of organic, inorganic or hybrid organic–inorganic molecules, making the applications for these nanomaterials even broader. We previously reported that ALD coating of MWCNTs with aluminum oxide (Al_2_O_3_) reduced the ability of MWCNTs to stimulate the production of pro-fibrotic cytokines in cultured human THP-1 monocytic cells in vitro and reduced MWCNT-induced lung fibrosis in mice in vivo [9]. In the present study, we sought to examine the effect of ALD coating of MWCNTs with zinc oxide (ZnO) on the inflammatory and fibrogenic response in human monocytic cells in vitro and after delivery to the lungs of C57BL6 mice in vivo by oropharyngeal aspiration (OPA).

ZnO nanoparticles (ZnO NPs) have numerous applications such as UV protection, bactericidal activity, and incorporation into coatings [13]. ZnO-coated MWCNTs (Z-MWCNTs) are used for a variety of novel applications. For example, aligned Z-MWCNTs form a stable, but reversible, super-hydrophobic material [14]. In addition, ZnO is photocatalytic and Z-MWCNTs can be used as a filter to degrade toxic components of industrial effluents [15]. Z-MWCNTs have also been explored as a nanogenerator [16]. With such a broad range of applications there is significant potential for human exposure to Z-MWCNTs.

The cellular or pathological effects of ZnO-coating on MWCNTs have not been investigated. Herein we report that MWCNTs coated with ZnO by ALD enhanced acute lung inflammation in mice and dramatically increased IL-6 in bronchoalveolar lavage fluid (BALF) and IL-6 mRNA in lung tissue at one day post-exposure. Moreover, IL-6 mRNA levels were increased in heart and liver from mice exposed to Z-MWCNTs, indicating a systemic acute phase immune response. Z-MWCNTs also markedly increased IL-6 mRNA levels in THP-1 monocytes compared to uncoated MWCNTs (U-MWCNTs). Mice exposed to Z-MWCNTs were acutely symptomatic and exhibited lethargy and shivering during the first 24 h after exposure but regained asymptomatic behavior thereafter. No significant differences in pulmonary fibrosis were observed at 28 days among mice treated with Z-MWCNTs or U-MWCNTs. This study expands the understanding of surface termination on the in vivo pulmonary response by contrasting the increased acute phase response caused by ZnO ALD coating in the present study with decreased toxicity and reduced fibrosis observed previously upon ALD coating of MWCNTs with Al_2_O_3_ [9].

## Results

### ALD on MWCNTs creates a conformal layer of ZnO that modifies physical properties

**Fig. 1:**
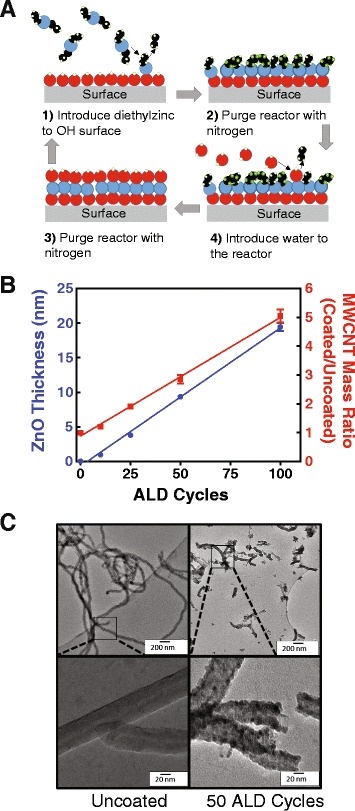
Atomic layer deposition (ALD) of ZnO on MWCNT. **a** Schematic of ALD on a generic surface. Reactants are introduced sequentially to build one self-limiting monolayer of material at a time. **b** ZnO thickness and material weight gain increase linearly with the number of ALD cycles. **c** TEM micrographs of MWCNT, uncoated and coated, show tube length decreases following sonication. Higher magnification images show conformal coating

Atomic layer deposition (ALD) utilizes a set of self-limiting reactions to create thin, conformal coatings (Fig. 1a). This can be accomplished at a range of temperatures and pressures to achieve the chosen coating [17]. Precursors, in this case diethyl zinc (DEZ) and water, were added one at a time and allowed to react with the surface. Between each step the ALD reactor was purged with nitrogen gas to remove all of the free chemical species. Cycles were repeated A B A B until the desired thickness was achieved. MWCNTs were coated with ZnO using ALD in a mesh basket surrounded by polypropylene to yield ZnO-coated MWCNTs (Z-MWCNTs). This allowed for the conformal coating of a high surface area, fine powder in a traditional ALD reactor. Diffusion through polypropylene and into the bulk MWCNT powder was achieved by allowing the precursors, DEZ and water, to remain in the reactor for a minute before purging. The resulting ZnO thickness was a function of ALD cycles where normal, linear ALD growth of roughly 2 Å/cycle was observed via measurements taken from TEM micrographs (Fig. 1b). The mass gain per cycle also showed a linear trend with mass increasing along with cycle number.

**Fig. 2:**
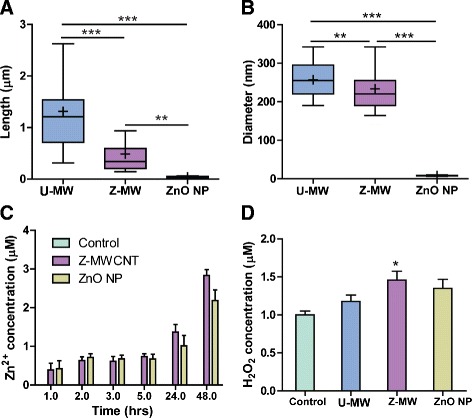
Characterization of uncoated MWCNTs (U-MW), ZnO-coated MWCNT (Z-MW), and ZnO nanoparticles (ZnO NP). **a** Length of ZnO coated MWCNT decreased post sonication as measured by TEM. **b** MWCNT aggregate size decreased with ZnO coating. For the data in panels A and B, The mean is represented as a + and the median as a solid line. For box and whisker plots the box represents the range from the 25th to 75th percentile. Significance is represented as * without a bracket as compared to the control, * denotes *P* < 0.05, ** denotes *P* < 0.01, and *** denotes *P* < 0.001. **c** Time course of Zn^+2^ ion concentration generated from Z-MWCNT or ZnO NP in serum free media. Zn^+2^ concentration was measured in serum-free medium in the absence of cells using 200 μg/ml of nanomaterial as described in Methods. Data are the mean and SEM of 5 measurements at each time point. **d** H_2_O_2_ production in serum free media was significantly increased by Z-MWCNT (Z-MW), but not U-MWCNT (U-MW), compared to control. Data are the mean and SEM of 6 replicate measurements. *P < 0.05 compared to control

We observed that the ZnO coating caused several changes in the physical and chemical properties of the MWCNTs. One of the most dramatic physical changes observed in this study caused by ALD coating with ZnO was the increased rigidity of these normally flexible MWCNTs. It was noted from TEM micrographs that the U-MWCNTs were longer than their coated counterparts (Fig. 1c). The Z-MWCNTs were completely coated with ceramic ZnO after 50 cycles, the cycle number used for these experiments. When these coated tubes were sonicated for 1 min to disperse them in solution, the rigid ceramic coating caused the Z-MWCNTs to break into shorter lengths; yet, very little delamination of the coating was seen and breakage was primarily noted at the tube ends (Fig. 1c). In contrast, sonication of U-MWCNTs for 1 min did not significantly affect tube length. Quantification of tube length showed that Z-MWCNTs were significantly shorter than U-MWCNTs after sonication (Fig. 2a). Z-MWCNT breakage upon sonication has also been observed with aluminum oxide coatings [9].

The dispersion of U-MWCNTs (40 μg/mL) was compared to Z-MWCNTs that had been coated with 50 cycles of ZnO as measured by dynamic light scattering in serum free media. A significant decrease in aggregate diameter was seen for Z-MWCNTs as compared to U-MWCNTs (Fig. 2b). This is likely due to the coating interrupting the Van der Walls interactions that normally occur between U-MWCNTs [18, 19].

As ZnO is slightly water-soluble there is potential for harmful concentrations of Zn^2+^ ions to leach from the Z-MWCNTs. The concentration of Zn^2+^ ions in serum free media from Z-MWCNT suspensions (200 μg/mL, all samples) was measured after 24 h incubation at 37 °C. There were detectable levels of Zn^2+^ in serum free media incubated with Z-MWCNTs or ZnO nanoparticles that increased in a time-dependent manner (Fig. 2c). Zn^+2^ ions were also measured in supernatants from THP-1 cells cultured for 24 h with either U-MWCNTs or Z-MWCNTs. A significant increase in Zn^+2^ was measured in the serum-free medium supernatants of THP-1 cells incubated for 24 h with Z-MWCNTs as compared to an equivalent amount of Z-MWCNTs incubated in serum-free medium in the absence of cells (Additional file 1).

Reactive oxygen species (ROS) were also measured in serum free media containing U-MWCNTs, Z-MWCNTs, or ZnO NPs (200 μg/mL, all samples) since ROS have been implicated as key players in MWCNT-induced pulmonary fibrosis [20]. It has been hypothesized that ZnO interacts with the cell membrane causing damage from electrostatic interaction or direct contact. We measured the hydrogen peroxide (H_2_O_2_) concentration after MWCNTs were incubated for 24 h at 37 °C in serum free media. Z-MWCNTs, but not U-MWCNTs, generated a significant increase in H_2_O_2_ compared to serum-free medium control (Fig. 2d).

As a point of comparison, ZnO nanoparticles (NPs) were also tested to determine how the presence of ZnO itself in the absence of MWCNTs would affect the inflammatory response in vitro ZnO NPs have the smallest diameter and aggregate size of all materials tested (Fig. 2a and b). ZnO NPs also showed a significant increase in Zn^2+^ concentration at approximately 1 μM, as compared serum-free medium control (Fig. 2c). ZnO NPs increased H_2_O_2_ above the serum-free media control, albeit not significantly (Fig. 2d).

### Z-MWCNTs stimulate pro-inflammatory cytokine expression by THP-1 cells *in vitro*

**Fig. 3:**
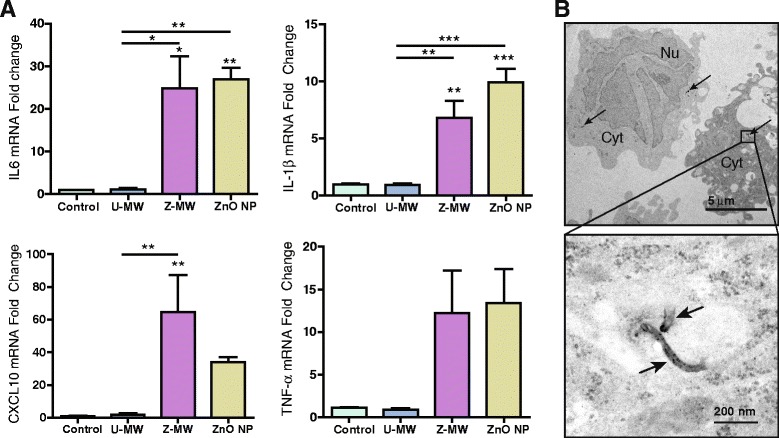
Cytokine mRNA expression in THP-1 cells 24 h after exposure to Z-MWCNTs, U-MWCNTs or ZnO NPs. **a** Taqman qRT-PCR was used to quantify IL-6, IL-1β, CXCL10 and TNF-α mRNA levels. Significant increases in pro-inflammatory cytokines were observed for both Z-MWCNT and ZnO NP treated cells. Asterisks represent comparisons to the control (*P < 0.05, **P < 0.01, and ***P < 0.001). Asterisks above a bar represent comparison to U-MWCNT. **b** Representative TEM images of THP-1 cells (upper panel) and U-MWCNT within cytoplasm (Cyt) of a THP-1 cell (lower panel). ‘Nu’ denotes nucleus. Arrows indicate U-MWCNTs within THP-1 cells. Z-MWCNTs were not observed within THP-1 cells

Human THP-1 cells were exposed to U-MWCNTs, Z-MWCNTs or ZnO NPs in vitro to develop predictive information on cytokine expression that could correlate with in vivo responses in mice. All nanomaterials were freshly prepared and sonicated 10 mg/mL stock solutions. Concentrations were as follows: Z-MWCNTs (40 μg/mL), U-MWCNTs (14 μg/mL) and ZnO NPs (26 μg/mL); where the concentration of U-MWCNTs was reduced to 14 μg/mL to normalize for nanoparticle number compared to Z-MWCNTs and ZnO NP concentrations were reduced to 26 μg/mL to match the total ZnO content of the Z-MWCNT samples, see Methods section for more details on this calculation. Cell viability determined by Trypan Blue staining demonstrated that the doses of Z-MWCNTs and ZnO NPs produced approximately 25 % cytotoxicity while the dose of U-MWCNTs did not cause a significant change in cytotoxicity (Additional file 2). Unprimed, non-adherent THP-1 cells were dosed and then collected 24 h later via centrifugation. Levels of IL-6, IL-1β and CXCL10 mRNAs were significantly increased for cells exposed to Z-MWCNTs compared to U-MWCNTs and control cells (Fig. 3a). ZnO NP exposure to THP-1 cells increased mRNA levels of IL-6 and IL-1β as compared to U-MWCNTs and control cells. CXCL10 mRNA was significantly increased with Z-MWCNT exposure but not by U-MWCNTs. TNF-α mRNA was also elevated for Z-MWCNTs and ZnO NP exposed cells, although not significantly. These trends matched those found in vivo one day after oropharyngeal aspiration with the exception of IL-1β, which was increased in THP-1 cells in vitro by Z-MWCNTs but not in the BALF of mice in vivo. Instead, U-MWCNTs increased IL-1β in the BALF of mice yet did not increase IL-1β in THP-1 cells in vitro. U-MWCNTs were found in the cytoplasm of THP-1 cells 24 h after treatment (Fig. 3b). In contrast, Z-MWCNTs were not observed within the cytoplasm of THP-1 cells (data not shown).

### ZnO coating enhances the acute lung inflammatory response to MWCNTs in mice

Mice were exposed to either U-MWCNTs or Z-MWCNTs via oropharyngeal aspiration at 4 mg/kg and 10 mg/kg, respectively, in 0.1 % pluronic saline solution so as to dose with the same number of MWCNTs, see Methods section for more details on dosing. Control mice were exposed to 0.1 % pluronic saline solution alone. Z-MWCNTs were coated with 50 ALD cycles to achieve a thickness of approximately 10 nm. To ensure that the same number of tubes was delivered for U-MWCNTs and Z-MWCNTs, the mass gain from ALD was used to calculate a normalized dose using the linear relationship shown in Fig. 1b. As such, Z-MWCNTs were dosed at 2.5 times that of U-MWCNTs since 60 % of the mass of Z-MWCNTs was due to the ZnO coating.

**Fig. 4:**
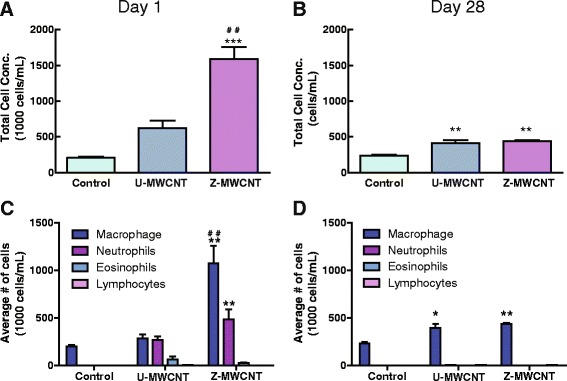
Cell counts in the BALF of mice exposed to U-MWCNTs or Z-MWCNTs. **a** The total cell concentration was significantly increased one day after exposure to Z-MWCNT as compared to control and U-MWCNT exposed mice, **b** A significant increase in total cell concentration was also seen at 28 days for U-MWCNTs and Z-MWCNTs. **c** After exposure to Z-MWCNTs, macrophages and neutrophils were significantly increased as compared to control. **d** Elevated levels of macrophages were seen 28 days after U-MWCNT exposure. Macrophages and neutrophils were still elevated 28 days after Z-MWCNT exposure. The number of animals per group at one day was: Control (3), U-MWCNT (4), Z-MWCNT (4) and at 28 days was: Control (4), U-MWCNT (5), Z-MWCNT (3). Significance is represented as * as compared to the control and # as compared to U-MWCNT, * denotes *P* < 0.05, ** or ^##^ denotes *P* < 0.01, and *** denotes *P* < 0.001

At 24 h post-exposure half of the mice in the study were euthanized via i.p. pentobarbital overdose followed by collection of BALF from the lungs. Mice exposed to U-MWCNTs showed a 3-fold increase in total number of cells in BALF compared to control at one day post-exposure (Fig. 4a). Mice exposed to Z-MWCNTs showed a significant and robust increase in total number of cells in BALF at one day post-exposure, a greater than 7.5-fold increase compared to control and a 2 to 3-fold increase above that observed for U-MWCNT treatment (Fig. 4a, c). By 28 days post-exposure, total cell counts in BALF were reduced compared to one day post-exposure, yet both U-MWCNT and Z-MWCNT exposure groups had significantly elevated total cell counts at 28 days compared to controls (Fig. 4b). Differential cell counting was performed to identify relative percentages of monocyte/macrophages, neutrophils, eosinophils and lymphocytes in BALF at one and 28 days. The majority of the cells present at one day after U-MWCNT exposure were an approximately equal mixture of macrophages and neutrophils, while cells increased by Z-MWCNTs exposure were represented by twice as many macrophages as compared to neutrophils (Fig. 4c). The BALF macrophages increased by either U-MWCNTs or Z-MWCNTs were most likely infiltrating monocytes that mature into alveolar macrophages, yet we did not differentiate between monocytes and macrophages when performing BALF cell counts. The majority of cells found 28 days after exposure were macrophages/monocytes for both treatment groups (Fig. 4d).

### ZnO coating prevents MWCNT uptake in the lungs of mice

**Fig. 5:**
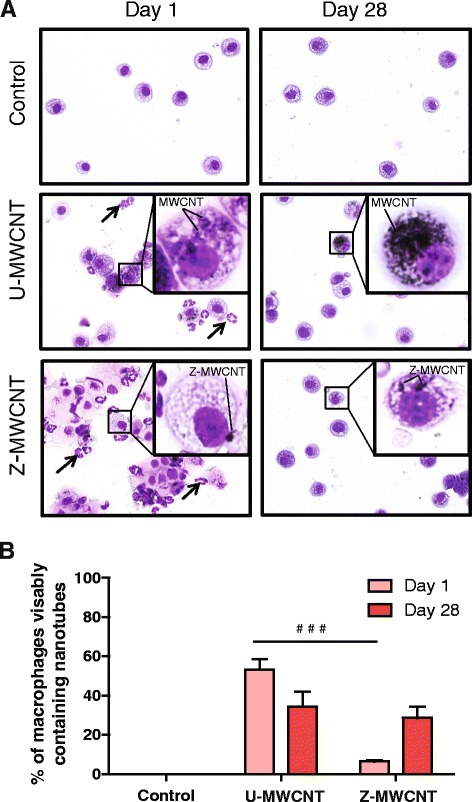
**a** Micrographs of cell populations from BAL fluid showing U-MWCNTs, but not Z-MWCNTs, taken up by macrophages at one day post-exposure. Arrows represent neutrophils. **b** Quantification of U-MWCNTs or Z-MWCNTs within macrophages at one and 28 days. One day after nanotube exposure more than 50 % of the macrophage population of U-MWCNT-treated mice have visibly engulfed MWCNTs while only about 10 % of the macrophages present in the Z-MWCNT treated mice have visibly engulfed MWCNTs. This difference evens out by day 28. The number of animals per group at one day was: Control (3), U-MWCNT (4), Z-MWCNT (4) and at 28 days was: Control (4), U-MWCNT (5), Z-MWCNT (3). Significance is represented as ### (*P* < 0.001) between U-MWCNT and Z-MWCNT at one day post-exposure

The relative number of macrophages in BALF that engulfed U-MWCNTs or Z-MWCNTs at one and 28 days was quantified from slides containing BAL cells isolated by Cytospin centrifugation. Interestingly, at one day post-exposure more than half of the macrophages in the BALF of U-MWCNT-treated mice contained MWCNT aggregates that were visible by light microscopy, while only 10 % were visible from Z-MWCNT-treated mice. In contrast, by day 28 the number of macrophages visibly containing MWCNTs was approximately equal (30-40 %) between U-MWCNT and Z-MWCNT treatments groups (Fig. 5).

### ZnO coating of MWCNTs increases the acute phase lung inflammatory response in mice but does not affect the chronic pulmonary fibrotic response

**Fig. 6:**
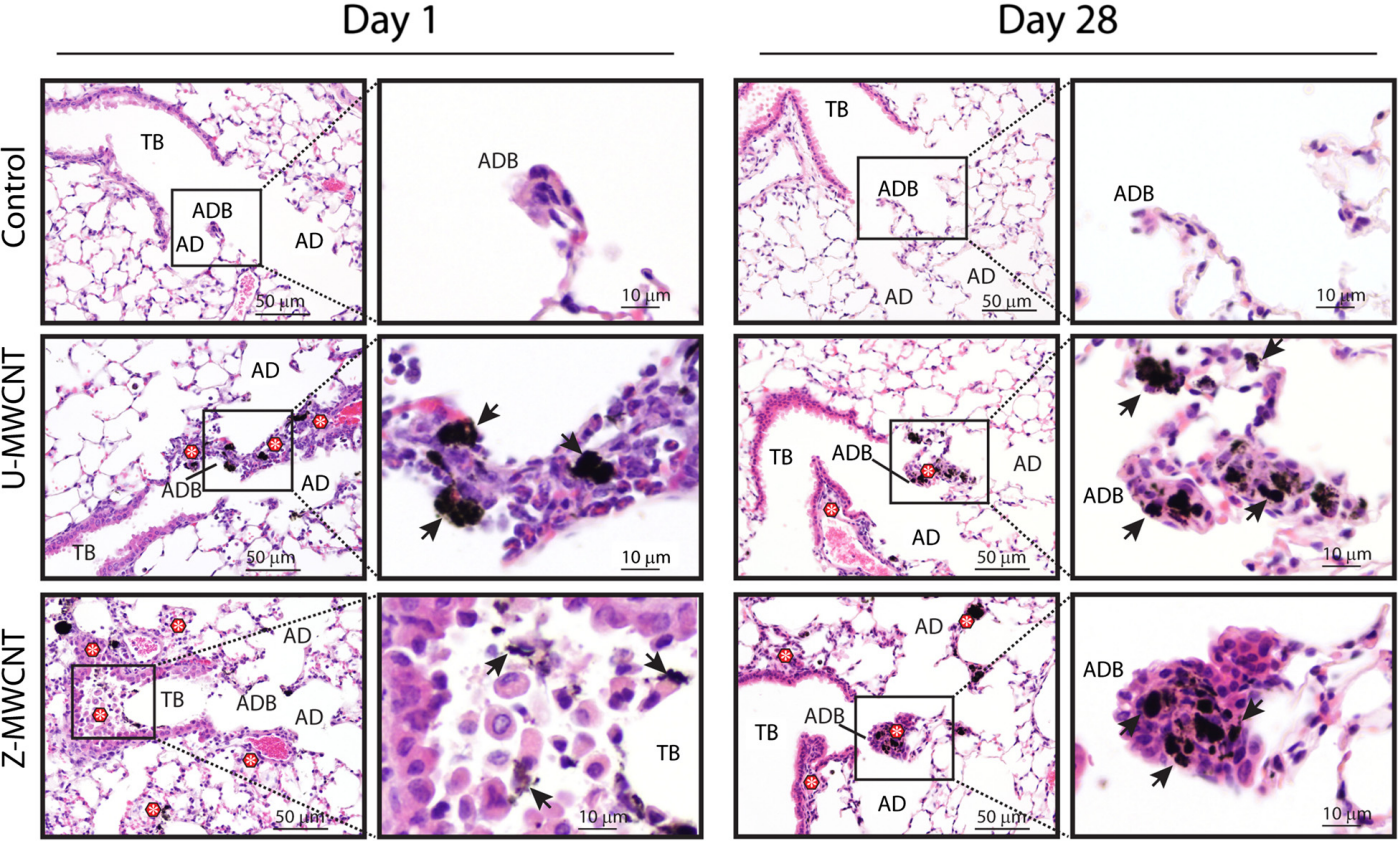
Histopathology of hematoxylin and eosin-stained mouse lung sections after exposure to U-MWCNTs or Z-MWCNTs at one and 28 days post-exposure. Treatment with U-MWCNTs caused more focal, condensed lesions at alveolar duct bifurcations (ADB) at day one (arrows) whereas Z-MWCNTs caused a more diffuse inflammatory response in the lower lung around terminal bronchioles (TB), ADB and alveolar ducts (AD). At day 28, both U-MWCNTs and Z-MWCNTs caused focal lesions at ADB (arrows). (*) indicate sites of inflammation

At day one and 28, lungs from U-MWCNT and Z-MWCNT-exposed mice were fixed and stained with hematoxylin and eosin or Masson’s trichrome stain to evaluate inflammation and fibrosis, respectively. As shown in Fig. 6, Z-MWCNTs produced a much more robust inflammatory response in the lungs of mice at one day post-exposure compared to U-MWCNT that largely resolved by day 28 in both treatment groups. The increase in inflammation observed by histopathological evaluation at one day post-exposure (Fig. 6) was consistent with the relative increases in BALF cell differential counts (Fig. 4). After 28 days, fibrotic lesions at alveolar duct bifurcations in lung sections stained with Masson’s trichrome appeared somewhat larger in mice exposed to Z-MWCNTs compared to mice treated with U-MWCNTs (Additional file 3). However, quantitation of total lung collagen by Sircol assay showed no significant changes in total lung collagen between treatment groups at either one or 28 days (Additional file 4).

### ZnO coating prevents MWCNT-induced DNA synthesis in airway epithelium of mice

**Fig. 7:**
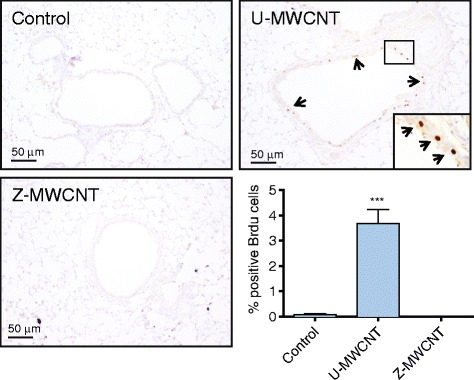
Bromodeoxyuridine (BrdU) labeled lung sections highlight airway epithelial cells undergoing DNA synthesis in treated and untreated mice. U-MWCNTs show a significant increase in the number of positively labeled cells as compared to both control and Z-MWCNT treated mice (inset panel shows BrdU positive cells indicated by arrows). The lower right hand panel shows quantitative data expressed as % positive BrdU airway epithelial cells relative to the total number of airway epithelial cells in cross-sectional profiles of small to medium-sized airways in lung sections. The number of animals per group at day one was: Control (3), U-MWCNT (4), Z-MWCNT (4). Significance is represented as *** (*P* < 0.001) U-MWCNTs compared to Control or Z-MWCNTs at day 1 post-exposure

DNA synthesis was measured in the lungs of mice exposed to U-MWCNTs or Z-MWCNTs as an index of lung cell proliferation using bromodeoxyuridine (BrdU) uptake. Airway epithelial cells were the only cells observed to be undergoing DNA synthesis in the lungs of U-MWCNT-exposed mice. BrdU-positive airway epithelial cells were quantified relative to the total number of airway epithelial cells in photomicrographs from control mice or mice exposed to U-MWCNTs or Z-MWCNTs. Less than 0.5 % of total lung cells were observed to uptake BrdU in the control 0.1 % pluronic saline group at one or 28 days. One day after exposure to U-MWCNTs, approximately 4 % airway epithelial cells were BrdU-positive, indicating cells undergoing DNA synthesis (Fig. 7). In contrast to mice exposed to U-MWCNTs, no BrdU uptake was observed in the airway epithelium of mice exposed to Z-MWCNTs.

### ZnO coating of MWCNTs increases pro-inflammatory cytokines in the lungs of mice

**Fig. 8:**
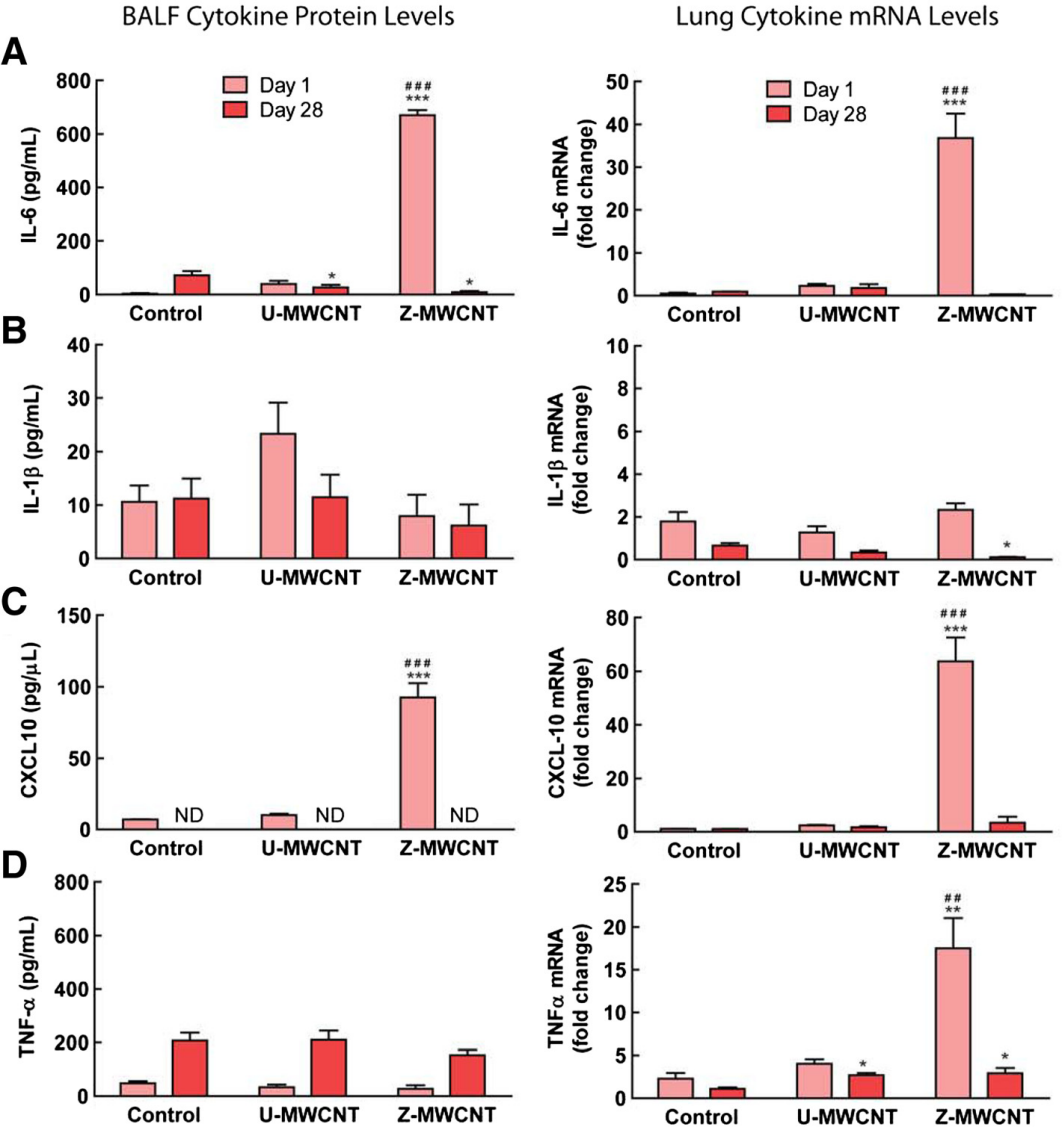
Lung cytokine protein and mRNA levels at one and 28 days post-exposure to U-MWCNTs or Z-MWCNTs. **a** IL-6, **b** IL-1β, **c** CXCL10 and **d** TNF-α protein and mRNA expression. in vivo lung exposure of mice to Z-MWCNTs elevated pro-inflammatory cytokine mRNA and protein levels one day after exposure while exposure to U-MWCNTs did not. Twenty-eight days later all levels are at or below control levels with the exception of TNF-α mRNA. The numbers of animals per group at day one was: Control (3), U-MWCNT (4), Z-MWCNT (4) and at 28 days was: Control (4), U-MWCNT (5), Z-MWCNT (3). Significance is represented as * as compared to the control and # as compared to U-MWCNT, * denotes *P* < 0.05, ** denotes *P* < 0.01, and *** denotes *P* < 0.001

Levels of cytokine proteins (IL-6, IL-1β, CXCL10, and TNF-α) were measured by ELISA in the BALF of mice following exposure to U-MWCNTs or Z-MWCNTs, and the corresponding cytokine mRNA levels were measured by Taqman real time RT-PCR in lung tissue. Z-MWCNTs markedly increased IL-6 protein and mRNA at one day post exposure in the lungs of mice and IL-6 levels returned to control levels by 28 days (Fig. 8a). In contrast, U-MWCNTs did not induce IL-6 protein or mRNA. IL-1β protein in BALF was increased approximately 2-fold, albeit not significantly, after exposure to U-MWCNTs, but was not increased by exposure to Z-MWCNTs (Fig. 8b). Z-MWCNTs significantly increased CXCL10 protein and mRNA at one day post-exposure and CXCL10 levels returned to control levels by 28 days (Fig. 8c). In contrast, U-MWCNTs did not induce CXCL10 protein or mRNA. Neither U-MWCNTs nor Z-MWCNT increased TNF-α protein levels in BALF from mice, yet TNF-α mRNA in lung tissue was significantly elevated by Z-MWCNTs at one day (Fig. 8d). There were no significant changes in the mRNA levels of the fibrotic mediator OPN in the lungs of mice exposed to U-MWCNT or Z-MWCNT at one or 28 days; mRNA levels of the fibrotic mediator TGF-β1 were significantly decreased by Z-MWCNT treatment compared to control (Additional file 5).

### Pulmonary exposure to ZnO-coated MWCNTs induces a systemic increase in IL-6 mRNA

**Fig. 9:**
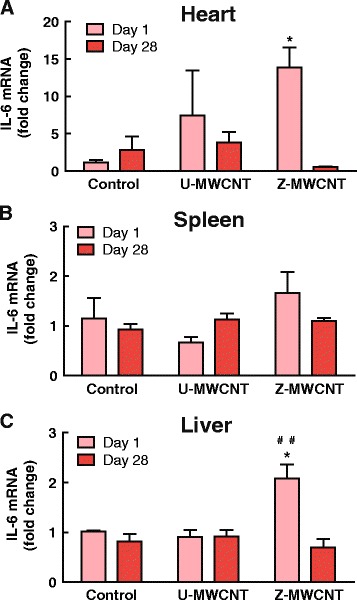
Systemic effects of Z-MWCNT exposure. **a** Expression of IL-6 in the heart is elevated significantly one day after lung exposure to Z-MWCNT. **b** IL-6 is slightly elevated in the spleen one day after Z-MWCNT exposure. **c** Z-MWCNT exposure caused significantly elevated IL-6 in the liver as compared to control and U-MWCNT dosed mice at one day. The number of animals per group at day one was: Control (3), U-MWCNT (4), and Z-MWCNT (4) and at 28 days was: Control (4), U-MWCNT (5), Z-MWCNT (3) . Significance is represented as * as compared to the control and # as compared to U-MWCNT, * denotes *P* < 0.05, ** denotes *P* < 0.01, and *** denotes *P* < 0.001

Since IL-6 is involved in the systemic acute phase response [21, 22] and because we observed a dramatic induction of IL-6 in lung tissue after Z-MWCNT exposure, we measured mRNA levels of IL-6 in heart, spleen and liver of mice exposed to U-MWCNTs or Z-MWCNTs. IL-6 mRNA was significantly increased in the heart and liver tissue of mice one day after exposure to Z-MWCNTs as compared to control mice (Fig. 9a, c). By day 28, IL-6 mRNA levels in heart and liver returned to control levels. A slight although not significant increase in IL-6 mRNA was also observed one day after treatment in the spleens of mice treated with Z-MWCNTs (Fig. 9b). Z-MWCNT-treated mice were symptomatic (lethargic and exhibited shivering) within the first 24 h after exposure, indicative of a systemic acute phase response, but returned to normal behavior by 48 h. In contrast, U-MWCNT-treated mice were not symptomatic. Of the five mice in the Z-MWCNT 28 day group, one died at day one and one at day six (Additional file 6).

## Discussion

As new applications for MWCNTs appear so do the number and types of functionalized MWCNTs, thus posing new and potentially unanticipated risks for human exposure. Investigations with rodents have already begun to address the biological consequences of MWCNT functionalization [5, 9, 23]. However, few studies evaluate how MWCNT functionalization by ALD surface modification or coating can influence lung disease in experimental animals in vivo. In previous work, we observed a reduced lung fibrotic response to Al_2_O_3_-coated MWCNTs (A-MWCNTs) compared to uncoated MWCNTs (U-MWCNTs) [9]. Moreover, the reduced lung fibrosis observed with A-MWCNTs corresponded to decreased BALF levels of OPN and TNF-α, both which play important roles in inflammation and fibrosis [3, 24]. However, while our previous work showed that A-MWCNTs caused less lung fibrosis than U-MWCNTs in mice at 28 days post-exposure, acute lung inflammation at one day post-exposure was not different between U-MWCNTs and A-MWCNTs [9]. The findings reported here with Z-MWCNTs contrast with previous findings on A-MWCNT toxicity in two ways. First, we show that Z-MWCNTs cause significantly greater inflammation compared to U-MWCNTs in the lungs of mice characterized by the infiltration of monocytes and neutrophils along with high levels of IL-6 and CXCL10. Second, the enhanced lung inflammation observed by Z-MWCNT at one day post-exposure did not result in changes in the amount of fibrosis at 28 days post-exposure. Fibrosis was similar between U-MWCNT and Z-MWCNT treatment groups as determined by histopathology and Sircol collagen assay but confined to different regions of the lung, likely due to differences in tube aggregation, length and density. Therefore, the findings reported here are novel because they emphasize that the specific chemical composition of surface coatings determine the nature of inflammatory and fibrotic responses caused by ALD-functionalized MWCNTs regardless of where the nanomaterial deposited in the lungs.

We observed a significant increase in IL-6 protein and mRNA in the lungs of mice one day after exposure to Z-MWCNTs, as well as elevated IL-6 mRNA levels in the heart and liver indicating a systemic immune response. IL-6 is a pleiotropic acute phase cytokine released in response to inflammatory stimulation and mediates cell proliferation, growth, differentiation, acute phase reactant production in the liver, and fever [21, 22, 25, 26]. Therefore, IL-6 is a likely candidate for mediating the dramatic pro-inflammatory response seen in the lungs of mice treated with Z-MWCNTs as well as the acute fever-like symptoms observed in mice in the first 24 h after exposure to Z-MWCNTs. CXCL10 was also highly increased in the lungs of mice after exposure to Z-MWCNTs compared to U-MWCNTs. CXCL10 is produced by monocytes in response to interferons secreted by T lymphocytes in response to pathogens in the body [27]. CXCL10 recruits monocytes, macrophages, Th1 lymphocytes, natural killer cells and dendritic cells [28]. Therefore, CXCL10 likely played a role in the high numbers of infiltrating monocytes observed in the BALF and lung tissue of mice treated with Z-MWCNTs at one day post-exposure. CXCL10 also plays an important role in tissue repair and has been shown to have an anti-fibrotic effect in vivo [29]. Therefore, the increased levels of CXCL10 in the lungs of mice exposed to Z-MWCNTs could have played a role in the resolution of the acute inflammatory response observed 1 day after Z-MWCNT exposure.

Alternative testing using in vitro cell culture models to predict biological responses in vivo has become increasingly important towards evaluating the toxicity of nanomaterials. The human THP-1 monocytic cell line was used in the current study to predict the inflammatory response to Z-MWCNTs in the lungs of mice in vivo. THP-1 cells are increasingly used to study the inflammatory or innate immune responses of macrophages to engineered nanomaterials [30, 31]. THP-1 cells are an appropriate cell culture model since circulating monocytes differentiate into macrophages after they infiltrate into lung tissue in response to an inflammatory stimulus and macrophages represent a first line of defense in the lungs by engulfing MWCNTs [3]. In the present study, IL-6 and CXCL10 mRNA expression in THP-1 cells in vitro induced by Z-MWCNTs closely matched the same pattern of induction for these two cytokines by Z-MWCNTs in the lungs of mice in vivo. TNF-α mRNA was induced in vivo in the lungs of mice by Z-MWCNTs only at the mRNA level, and while this same trend in TNF-α mRNA induction was observed in THP-1 cells in vitro, it was not statistically significant. However, Z-MWCNTs also increased IL-1β mRNA levels in THP-1 cells in vitro, but IL-1β was not induced by Z-MWCNTs in vivo. IL-1β is a cytokine released from macrophages and is a mediator of inflammation [32]. Therefore, our in vitro cytokine expression was only partly predictive of in vivo cytokine expression. The in vitro responses of THP-1 cells to MWCNTs can be modified by a variety of factors, including LPS priming and/or treatment with phorbol ester to differentiate monocytes to macrophages. In the present study, THP-1 cells were neither primed with LPS nor stimulated with phorbol ester and thus represented a monocyte phenotype.

Interestingly, U-MWCNT stimulated airway epithelial DNA synthesis in the airway epithelium of mice as measured by BrdU uptake, whereas no significant DNA synthesis was observed in the airway epithelium of Z-MWCNT-exposed mice. The airway epithelial proliferative response one day after U-MWCNT exposure is similar to the response observed in rats after inhalation exposure to chrysotile asbestos [33]. The incorporation of BrdU into airway epithelial cells following U-MWCNT exposure likely represents a response to injury where DNA synthesis and cell cycle progression are initiated to allow for epithelial cell proliferation as part of a homeostatic repair process. The lack of BrdU incorporation in airway epithelium after exposure to Z-MWCNT suggests that the ZnO coating or dissolution of Zn^+2^ ions causes epithelial cell growth arrest. Cell cycle arrest has been reported in RSC96 Schwann cells and in epidermoid cancer cells exposed to varying concentrations of ZnO NPs in vitro [34, 35]. Both of these previous studies cited the ability for ZnO NPs to increase ROS and thus induce DNA damage thereby halting DNA synthesis. H_2_O_2_ has recently been demonstrated as a primary mediator of Zn-induced oxidative stress in human airway epithelial cells [36]. The present study shows that Z-MWCNTs increased H_2_O_2_ in cell-free media. In addition, we detected Zn^+2^ ions after incubation of Z-MWCNTs in media, indicating some degree of dissolution. Therefore, it is possible that growth arrest of the airway epithelium in mice exposed to Z-MWCNTs is due a ROS-dependent mechanism involving dissolution of Zn^+2^ ions. Alternatively, ROS could be generated from the surface of Z-MWCNTs.

Previous studies have shown that Zn causes toxicity and lung injury through the release of Zn^+2^ or via ROS-dependent mechanisms. For example, the soluble fraction of combustion emission particulate matter mediates lung inflammation in rats and this is due in part to dissolution of metal ions, including Zn^+2^ [37]. In addition, occupational inhalation exposure of welders to Zn causes an acute lung inflammatory response referred to as “metal fume fever” and this is largely mediated via soluble Zn^+2^ [38]. Moreover, it has been shown that ZnO can generate ROS, specifically H_2_O_2_, via an aqueous phase reaction with oxygen vacancies within the ZnO crystal lattice [39]. Some evidence suggests that ROS do not have a large contribution to ZnO toxicity, citing that the antioxidant NAC commonly used in studies of ZnO-induced ROS generation is a chelator of zinc and is therefore only reducing cytotoxicity due to the sequestration of zinc ions in solution [40].

It is also a point of controversy as to where the ZnO is dissolving; inside or outside of cells. Xia and coworkers found that non-dissolved ZnO NPs were taken up by BEAS-2B epithelial cells in caveolae by fluorescently labeling the nanoparticles and staining for calveolin-1 [41]. That same study showed that uptake of ZnO NP by RAW 264.7 macrophages occurred in lysosomes that completely dissolved the nanoparticles [41]. Further work by these authors concluded that although there is some dissolution of ZnO in the media, the main contributor to dissolution is ZnO NP uptake and dissolution within the cell [42]. This is contrasted by a report from Buerki-Thurnherr and colleagues, wherein they concluded that dissolution primarily occurs in the media using Jurkat cells as they were unable to visualize any nanoparticles in the cells via TEM [40]. We have found that ZnO dissolves slowly in serum-free defined medium (SFDM) with a slow increase in the concentration of Zn^2+^ seen between one and 48 h (Fig. 2c). Additionally, we have observed that when dissolution in SFDM alone was compared to dissolution in SFDM with THP-1 cells present the samples with cells had concentrations almost 7 times higher than the samples without cells (Additional file 1). However, as discussed below, Z-MWCNTs were not avidly taken up by phagocytes in vitro or in vivo. Therefore, enhanced Zn^+2^ dissolution from Z-MWCNTs in the presence of cells apparently does not require cellular uptake.

Finally, our data show that Z-MWCNTs evaded macrophage uptake in the lungs of mice at one day post-exposure and in THP-1 cells in vitro, whereas U-MWCNTs were avidly engulfed by macrophages in vivo and by THP-1 cells in vitro. The reason for evasion of macrophage uptake by Z-MWCNTs remains unclear and requires further investigation. However, Z-MWCNTs not taken up by macrophages would have a greater opportunity to interact with the lung epithelium and cause toxicity. Collectively, our data suggest that the ZnO coating on Z-MWCNTs causes airway epithelial growth arrest through the release of H_2_O_2_ and this could be due either to release of Zn^+2^ ions through dissolution or through direct interaction of the surface of Z-MWCNTs with epithelial cell membranes at the nano-bio interface.

In addition to changing the surface chemistry, the ZnO coating also changes the length and aggregation of the Z-MWCNTs. ZnO is a brittle ceramic material. Once coated by ALD, Z-MWCNTs break into smaller, more dispersed segments after sonication. Agglomeration of MWCNTs is due to Van der Waals interactions, making them difficult to disperse [18, 19]. By coating the tubes there is the potential to interrupt these interactions. Previous studies have suggested that both MWCNT dispersal state and length play a role in inducing fibrosis. Agglomerated MWCNTs produce granulomas in the lungs of rodents, while dispersed MWCNTs lead to diffuse interstitial pulmonary fibrosis [5, 25, 43]. MWCNT length also influences cellular response and toxicity. For example, long, rigid materials lead to frustrated phagocytosis by macrophages, resulting in lysosomal membrane damage and release of ROS and pro-inflammatory cytokines [1]. Moreover, decreasing fiber or tube length (i.e., aspect ratio) results in decreased toxicity and more rapid clearance from lung tissue [44, 45]. In the present study, we found that shorter and better dispersed Z-MWCNTs caused more acute inflammation than U-MWCNTs. While the acute phase immune response to Z-MWCNTs observed in this study was likely mediated by surface ZnO and generation of H_2_O_2_, greater dispersal of nanotubes could also play a role by increasing bioavailability in the lungs of exposed animals.

In a broader context, the present study extends a growing literature showing that functionalization of MWCNTs can alter biological responses and thereby potentially pose unanticipated hazards for human exposure. However, unlike the enhanced toxicity and immunogenic reactions seen with Z-MWCNTs compared to U-MWCNTs in the present investigation, other studies have shown that certain types of functionalization can decrease the toxic response to MWCNTs. For example, COOH-functionalized MWCNTs induced less lung inflammation and reduced fibrosis in the lungs of mice compared to pristine MWCNTs [5, 23]. As mentioned previously, Al_2_O_3_ coating of MWCNTs applied by ALD reduced lung fibrogenesis [9]. Therefore, with regards to ALD functionalization, the chemical composition of the thin-film coating determines lung toxicity and pathologic outcome. Since novel applications of ZnO-coated MWCNTs are increasing in diversity [14–16], our work in the present study has important human health implications for exposure to these functionalized nanomaterials.

## Conclusions

**Fig. 10:**
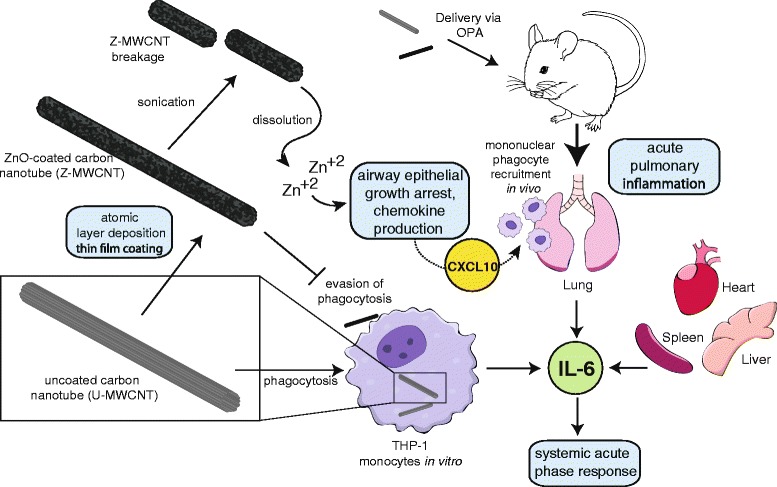
Illustration of proposed mechanisms underlying the acute phase immune response to ZnO-coated MWCNTs (Z-MWCNTs) in human THP-1 monocytes in vitro and after delivery to the lungs of mice *in vivo* by oropharyngeal aspiration. Uncoated MWCNTs (U-MWCNTs) are subjected to atomic layer deposition (ALD) coating with ZnO to yield Z-MWCNTs. Sonication results in breakage of Z-MWCNTs and the ZnO coating also undergoes partial dissolution to release Zn^+2^ ions in aqueous media. Unlike U-MWCNTs, Z-MWCNTs are not taken up by phagocytic monocytes or macrophages in vitro or in vivo. Z-MWCNTs cause epithelial cell growth arrest and increases in CXCL10, a monocyte chemoattractant, as well as increases in IL-6 that mediates systemic acute phase responses

In summary, we report that functionalization of MWCNTs by atomic layer deposition (ALD) with ZnO increased pro-inflammatory cytokine expression by THP-1 monocytic cells in vitro and caused an acute phase lung and systemic immune response in mice one day after exposure by oropharyngeal aspiration. Interestingly, pulmonary fibrosis induced by Z-MWCNT at 28 days post-exposure was not significantly different from that seen with U-MWCNTs. As illustrated in Fig. 10, the mechanism underlying the acute phase inflammatory response to Z-MWCNTs involves dissolution of Zn^+2^ ions from the ZnO coating, H_2_O_2_ generation, growth arrest of airway epithelial cells, and induction of CXCL10 and IL-6. These observations contrast with previous findings of reduced lung toxicity and fibrosis by MWCNTs functionalized by ALD-coating with Al_2_O_3_ and emphasize the importance of chemical composition as a primary factor of the pulmonary innate immune response to ALD-functionalized MWCNTs.

## Methods

### Chemicals and materials

Diethylzinc (DEZ) (Strem Chemicals, min 98 % pure) was used as received. DEZ was co-reacted with deionized (DI) water. The reactor was purged with high purity nitrogen gas (Machine & Welding Supply Co) that was further purified with a Entegris GateKeeper located directly upstream from the reactor input. Silicon substrates (University Wafers, P-type, <100>) were used to monitor the growth of zinc oxide. Multi-walled carbon nanotubes (MWCNT) (Helix Materials Solutions, 0.5-40 um in length) were coated as received. Zinc oxide nanoparticles (ZnO NP) (UC CEIN) were used as a positive control as received.

### MWCNT Atomic Layer Deposition (ALD)

MWCNTs were coated utilizing a method previously described [10]. Briefly, approximately 30 mg of MWCNTs were placed into a mesh cylinder surrounded by a nonwoven polypropylene (PP) sheet (melt-blown, NC State University, College of Textiles) and secured using white, 100 % cotton thread. The PP sheet was measured so as to minimize material overlap and promote diffusion of atomic layer deposition (ALD) precursors. A silicon wafer monitor was placed upstream of the encased MWCNTs and similarly wrapped. Behind the MWCNTs was placed an unwrapped silicon wafer monitor. Samples were placed into a custom made, viscous-flow, hot-walled, vacuum reactor [46–48]. The reactor was kept at roughly 800 mTorr, and operated at 35 °C. DEZ was introduced into the reactor and held by closing all ports into and out of the reactor for 60 s; this allowed for proper diffusion of the precursor through the PP. The reactor was then purged with N_2_ gas. This was followed by a co-reacting step with DI water that was also allowed to be held in the reactor for 60 s.

### MWCNT characterization

ZnO coating thickness was determined by spectroscopic ellipsometry (J.A. Woollam Co., Inc) of the monitor silicon wafers. Thickness was also measured using a JEOL 2000FX scanning transmission electron microscope (TEM). TEM samples were prepared by dropping 3 μl of ENM (engineered nanomaterials) suspended in 100 % ethanol on to a carbon faced TEM grid (Protochips) and allowing the suspension to dry. Samples were sonicated using the method described above before TEM grid preparation. From TEM images the length of the ENM was measured. ImageJ software was used to measure MWCNT length and ZnO thickness.

Mass gain of the MWCNT after the ALD coating process was measured (Fischer Scientific, accuSeries-accu124) [10]. This allowed for validation that the nanomaterial was being coated, and also for the potential to correct for this weight change when dosing to normalize to the number of MWCNT dosed instead of the total weight.

Dynamic light scattering (DLS, Malvern Zetasizer ZSP) was used to determine the MWCNT aggregate size. ENM were suspended as described above and then diluted to a concentration of 40 μg/mL in serum free media, the same that was used to serum starve cells. Following one hour of settling, shorter times resulted in inconsistent data as large aggregates actively settled, the samples were measured. Values were reported using the number percent of the diameter measured. Three different samples were used to establish significance.

Zn^2+^ concentrations were measured with a NanoMolar Zinc Assay Kit (ProFoldin) according to manufacturer’s instructions. ENM were incubated in serum free media for 24 h in the dark at 37 °C at a concentration of 200 μg/mL. Florescence was read using a FLUOstar Omega (BMG Labtech).

H_2_O_2_ concentrations were measured using an Amplex Red Assay (Thermo Fisher Scientific) according to the manufacturer’s instructions. U- and Z-MWCNTs or ZnO nanoparticles were incubated in serum free media for 24 h in the dark at 37 °C at a concentration of 200 μg/mL. Absorbance was read at 560 nm using a microplate spectrophotometer (Multiskan EX, ThermoFisher Scientific).

### Cell culture and dosing

Human monocytes (THP-1) (ATCC) were cultured in RPMI-1640 medium (Life Technologies) supplemented with 10 % fetal bovine serum (Gibco) and kept at 37 °C with 5 % CO_2_. Cells were transferred into 35 mm plates (Becton Dickinson) for experimentation at a concentration of approximately 4.5x10^5^ cells/mL. Once plated, cells were serum starved in F-12 K Nutrient Mixture with albumin solution and ITS (Gibco, Sigma- 35 % in DPBS, and Lonza respectively) overnight. The concentration range of MWCNT used dosing THP-1 cells in vitro was consistent with that previously established through intra-laboratory consortium testing of engineered nanomaterials [49]. Cells were dosed with a stock solution (10 mg/mL) of engineered nanomaterials (ENM, in this case MWCNT that were either uncoated or coated in ZnO as well as ZnO NP) in sterile 0.1 % pluronic F-68 (Sigma-Aldrich). The dose of U-MWCNTs, Z-MWCNT and ZnO NP were 14, 40 and 26 μg/mL, respectively. The Z-MWCNT dose was normalized to achieve a dose with as similar a number of nanoparticles as possible to the uncoated tubes. To adjust this the mass gain following ALD was utilized in Eq. 1:

1 $$ MWCNTdose\ast \frac{m_{coatedMWCT}}{m_{uncoatedMWCNT}}=Z-MWCNTdose $$

Where “m” is the mass of the nanomaterial in grams and the dose is in μg/mL. In this case the value for the mass of coated MWCNT divided by the mass of uncoated MWCNT for this experiment was 2.8 and thus the 14 μg/mL U-MWCNT dose corresponds to a 40 μg/mL Z-MWCNT dose. The ZnO NP dose was such that the total mass of ZnO in the Z-MWCNT dose and the ZnO NP dose was equivalent. To calculate this the following equation was used:

2 $$ ZnONPdose=Z-MWCNTdose\ast \frac{m_{coatedMWCNT}-{m}_{uncoatedMWCNT}}{m_{coatedMWCNT}} $$

In this case the ratio between the difference of the coated and uncoated tubes to the coated tubes was 0.65 corresponding to 65 % of the coated tubes being comprised of ZnO. Vials of ENM were suspended using a cuphorn sonicator (Qsonica) at room temperature immediately preceding dosing using 7 amps, 50 W for a total energy of 2910 J on average.

### Cell viability

Cell viability was determined using a 0.4 % Trypan Blue solution (Life Technologies) according to the manufacturer’s protocol. Briefly, Trypan Blue was mixed 1:1 with the THP-1 cell suspension and the number of living and dead cells was counted using a hemocytometer.

### Mouse exposure to MWCNTs

All animal procedures were approved by the NC State University Institutional Animal Care and Use Committee (Protocol #13-086-B). C57BL6 mice (Jackson Laboratories) were exposed to U-MWCNTs or Z-MWCNTs (50 ALD cycles) suspended in 0.1 % pluronic saline solution via oropharyngeal aspiration under isoflurane anesthesia at 4 mg/kg and 10 mg/kg, respectively, in order to deliver the same number of MWCNTs per mouse. The control group of mice was exposed to pluronic alone. Each treatment group (Control, U-MWCNT, Z-MWCNT) contained 3, 4 and 4 animals at one day, respectively, and 4, 5, and 5 animals at 28 days, respectively. To ensure that the same number of tubes was delivered for U-MWCNTs and Z-MWCNTs, the mass gain from ALD was used to calculate a normalized dose. To do this calculation the same approach was used as in the above section. In this case the batch of Z-MWCNT used had a value of 2.5 for the ratio of mass of coated MWCNT/mass of uncoated MWCNT. As such, Z-MWCNTs were dosed at 2.5 times that of U-MWCNTs for a total of 10 mg/kg. At day one and 28 after exposure, mice were euthanized via intraperitoneal injection of pentobarbitol (Fatal Plus, Vortech Pharmaceuticals). Bronchoalveolar lavage fluid (BALF) was collected from the lungs via two serial lavages of 0.5 mL of phosphate buffered saline (PBS, Dulbecco) and combined. BALF was used to determine cells/mL, cell type and protein content via enzyme-linked immunosorbent assay (ELISA). The caudal and middle lobes of the right lung were stored in RNAlater (Ambion) and used to determine mRNA profiles (as well as heart, liver and spleen). The left lungs were fixed for 24 h using 10 % neutral buffered formalin via intratracheal infusion and then transferred to 70 % ethanol. The left lung was then embedded in paraffin, sectioned and stained with hematoxylin and eosin (H&E) or Masson’s trichrome and imaged using an Olympus BX40 light microscope.

### Bromodeoxyuridine (BrdU) Immunohistochemistry

For BrdU incorporation, each animal received an intraperitoneal injection of 100 mg/kg from a stock of 10 mg/mL BrdU (Sigma #B5002) in PBS 1 h prior to sacrifice. Paraffin blocks were cut 5 μm with a microtome and mounted on a negatively charged slide and dried overnight. The sections were then hydrated and immunostained with anti-BrdU Pure (BD#347580) followed by the vectastain ABC kit (VectorLabs#PK-6102) and DAB buffer (BioGenex#HK542-XAK) as described per manufacturer inserts. The positive brown cells uniquely stood out from the hematoxalin counterstain. Quantification of BrdU positive cells was achieved by counting the number of BrdU positive cells as well as the total number of cells per airway. All of the airways from each mouse for each treatment were combined and data was reported as a percentage of BrdU positive cells per treatment group.

### Sircol collagen assay

Soluble collagen in lung tissue was measured by Sircol assay (Biocolor, Carrickfergus, UK) according to the manufacturer’s instructions.

### Cytology

Cell concentrations from the BALF were determined via hemocytometer. A Cytospin 4 (Thermo-Fisher Scientific) was used to deposit cells from the BALF onto glass slides. Cells were then fixed and stained using a Diff-Quik Stain Set (Siemens). Relative percentages of macrophages, neutrophils, eosinophils, or lymphocytes per 500 cells were then identified using a light microscope. The percent of macrophages visually containing U-MWCNTs or Z-MWCNTs per 100 cells per mouse was also determined.

### RT-PCR

SuperScript^(R)^ III Platinum One-Step qRT-PCR system (Life Technologies) was used in conjunction with a StepOnePlus Real-Time PCR System (Applied Biosystems) to determine the fold change of mRNA for IL-6, IL-1β, CXCL10, TNF-α, OPN, and TGF-β1. RNA was extracted from homogenized lung, heart, liver and spleen tissues using Quick-RNA™ MiniPrep (Zymo Research) according to the manufacturer’s instructions. 18S was used as an endogenous control “housekeeping gene” for all in vitro experiments. THP-1 cells were collected from suspension via centrifugation for 5 min at 1000 rpm. B2M was used as the endogenous control for all mouse experiments.

### ELISA

IL-6, IL-1β, CXCL10, TNF-α, OPN, and TGF-β1 protein levels in the BALF were measured via ELISA (DuoSet, R&D Systems). Samples were assayed according to manufacturer instructions. Absorbance was read at 450 nm by a microplate spectrophotometer (Multiskan EX, ThermoFisher Scientific) with a correction wavelength of 540 nm.

### Data and statistical analysis

Data and statistical analysis was performed using GraphPad Prism version 5.0 (GraphPad Software Inc.). A one-way ANOVA with a *post hoc* Tukey test was used to determine significance between samples. A significance of *p* < 0.05 was used unless otherwise noted. Data values were expressed as mean ± SEM.

## Abbreviations

ALD, atomic layer deposition; BALF, bronchoalveolar lavage fluid; BrdU, bromodeoxyuridine; OPA, oropharyngeal aspiration; U-MWCNT, uncoated multi-walled carbon nanotube; Z-MWCNT, ZnO-coated multi-walled carbon nanotube; ZnO, zinc oxide.

## Additional files

Additional file 1: Zn^+2^ ion concentration in serum-free defined medium after exposure to U-MWCNTs or Z-MWCNTs in the absence or presence of THP-1 cells. (PDF 315 kb)

Additional file 2: THP-1 cell viability after exposure to U-MWCNTs or Z-MWCNTs. (PDF 423 kb)

Additional file 3: Trichrome-stained lung sections from mice after exposure to U-MWCNTs or Z-MWCNTs. (PDF 959 kb)

Additional file 4: Collagen measurement in the lungs of mice exposed to U-MWCNTs or Z-MWCNTs. (PDF 181 kb)

Additional file 5: TGF-β1 and OPN mRNA from the lungs of mice exposed to U-MWCNTs or Z-MWCNTs. (PDF 206 kb)

Additional file 6: Mouse survival after exposure to U-MWCNTs or Z-MWCNTs. (PDF 298 kb)
