# Supplementary material for: Atomic layer deposition coating of carbon nanotubes with zinc oxide causes acute phase immune responses in human monocytes in vitro and in mice after pulmonary exposure
Source: Part Fibre Toxicol. 2016 Jun 8;13:29. doi: 10.1186/s12989-016-0141-9 (PMC4899913; doi:10.1186/s12989-016-0141-9)
Supplement: Supplementary file 1 — Zn+2 ion concentration in serum-free defined medium after exposure to U-MWCNTs or Z-MWCNTs in the absence or presence of THP-1 cells. (PDF 315 kb) [file 12989_2016_141_MOESM1_ESM.pdf]

Additonal File 1

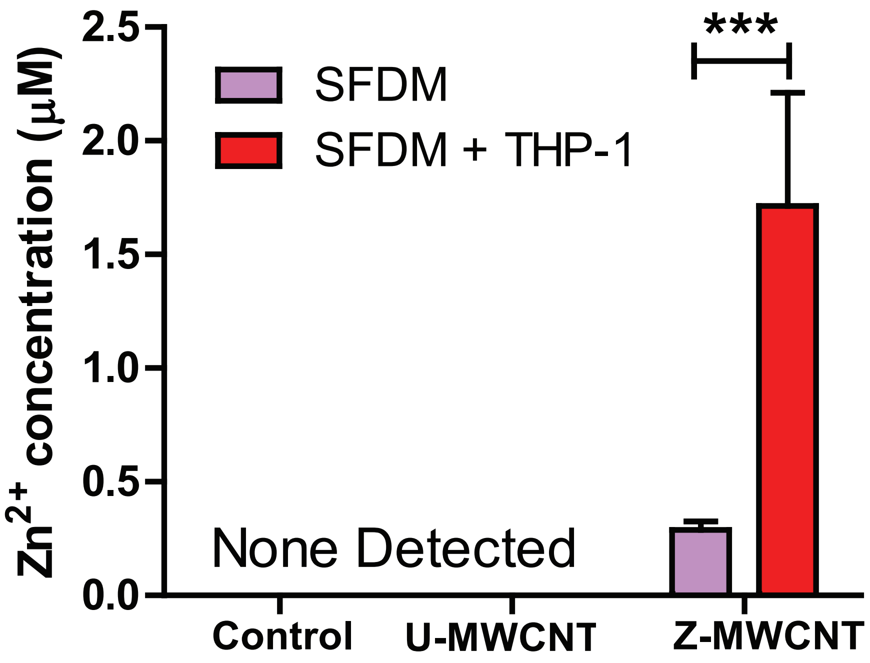

Additonal File 1. Zn^{+2} ion concentration in serum-free defined medium (SFDM) incubated with uncoated MWCNT (U-MWCNT) or ZnO-coated MWCNT (Z-MWCNT) in the absence of cells or in SFDM from THP-1 cells incubated with U-MWCNTs or Z-MWCNTs (SFDM + THP-1). 40 μg/ml of Z-MWCNTs or 14 μg/ml U-MWCNT were dosed into SFDM with or without THP-1 cells for 24 hrs. Data are the mean $\pm$ SEM of 6 replicate determinations.
