# Supplementary material for: Atomic layer deposition coating of carbon nanotubes with zinc oxide causes acute phase immune responses in human monocytes in vitro and in mice after pulmonary exposure
Source: Part Fibre Toxicol. 2016 Jun 8;13:29. doi: 10.1186/s12989-016-0141-9 (PMC4899913; doi:10.1186/s12989-016-0141-9)
Supplement: Supplementary file 2 — THP-1 cell viability after exposure to U-MWCNTs or Z-MWCNTs. (PDF 423 kb) [file 12989_2016_141_MOESM2_ESM.pdf]

Additional File 2

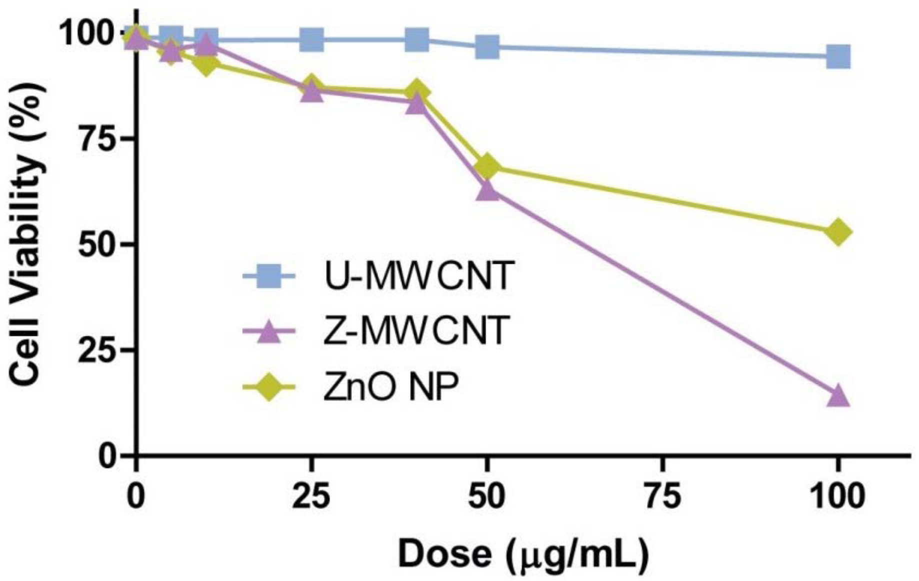

Additional File 2. Dose-dependent decrease in cell viability of THP-1 cells 24 hrs after exposure to U-MWCNTs, Z-MWCNTs, or ZnO nanoparticles (NP). Cell viability was measured by Trypan Blue staining. Data represent the average of living cells from a total of 100 to 300 cells per dose and treatment.
