# Supplementary material for: Atomic layer deposition coating of carbon nanotubes with zinc oxide causes acute phase immune responses in human monocytes in vitro and in mice after pulmonary exposure
Source: Part Fibre Toxicol. 2016 Jun 8;13:29. doi: 10.1186/s12989-016-0141-9 (PMC4899913; doi:10.1186/s12989-016-0141-9)
Supplement: Supplementary file 3 — Trichrome-stained lung sections from mice after exposure to U-MWCNTs or Z-MWCNTs. (PDF 959 kb) [file 12989_2016_141_MOESM3_ESM.pdf]

Additional File 3

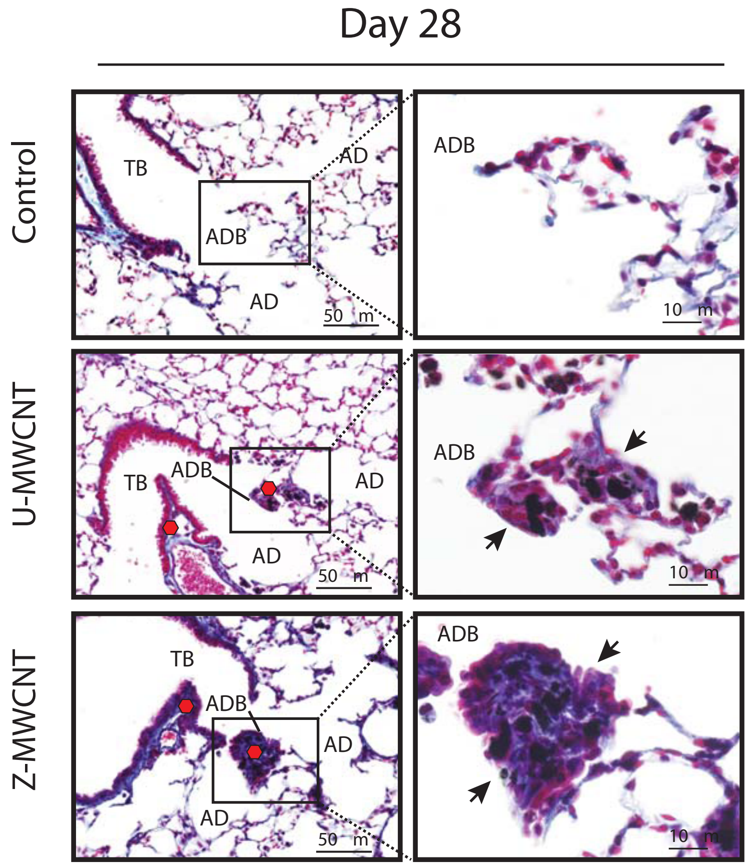

Additional File 3. Lung sections stained with Masson's trichrome from mice 28 days after exposure to saline pluronic (Control), U-MWCNTs or Z-MWCNTs. Collagen is indicated by blue stain (arrows). TB (termial bronchiole), ADB (alveolar duct bifurcation), AD (alveolar duct).
