# Supplementary material for: Atomic layer deposition coating of carbon nanotubes with zinc oxide causes acute phase immune responses in human monocytes in vitro and in mice after pulmonary exposure
Source: Part Fibre Toxicol. 2016 Jun 8;13:29. doi: 10.1186/s12989-016-0141-9 (PMC4899913; doi:10.1186/s12989-016-0141-9)
Supplement: Supplementary file 4 — Collagen measurement in the lungs of mice exposed to U-MWCNTs or Z-MWCNTs. (PDF 181 kb) [file 12989_2016_141_MOESM4_ESM.pdf]

Additonal File 4

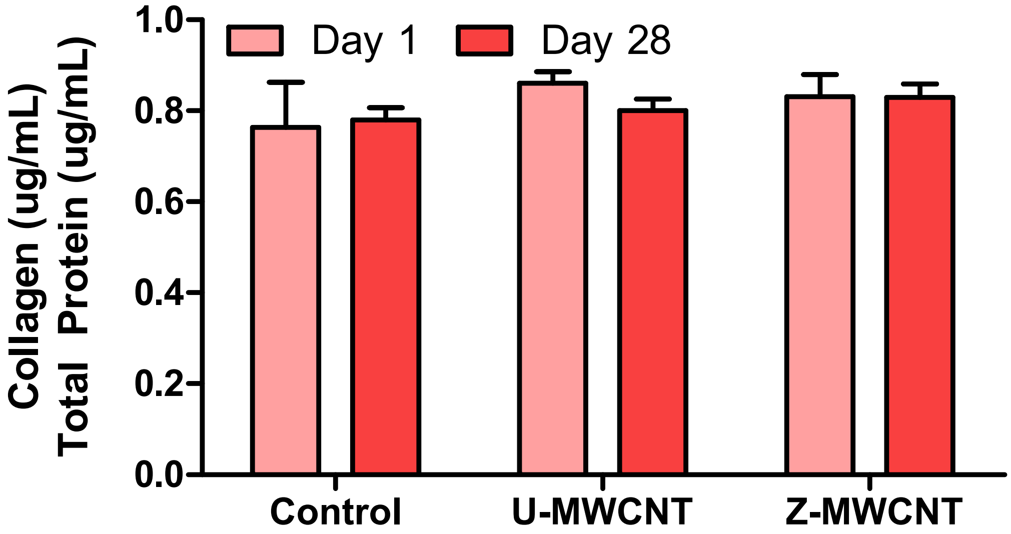

Additonal File 4. Lung collagen levels in mice following oropharyngeal aspiration of Z-MWCNTs or U-MWCNTs. Right lung lobes were collected at 1 and 28 days and collagen measured by Sircol assay. Collagen levels were normalized to total lung protein in each sample. Each treatment group (Control, U-MWCNT, Z-MWCNT) contained 3, 4 and 4 animals at one day, respectively, and 4, 5, and 5 animals at 28 days, respectively. No significant differences were observed between treatment groups at either time point.
