# Supplementary material for: Atomic layer deposition coating of carbon nanotubes with zinc oxide causes acute phase immune responses in human monocytes in vitro and in mice after pulmonary exposure
Source: Part Fibre Toxicol. 2016 Jun 8;13:29. doi: 10.1186/s12989-016-0141-9 (PMC4899913; doi:10.1186/s12989-016-0141-9)
Supplement: Supplementary file 6 — Mouse survival after exposure to U-MWCNTs or Z-MWCNTs. (PDF 298 kb) [file 12989_2016_141_MOESM6_ESM.pdf]

Additional File 6

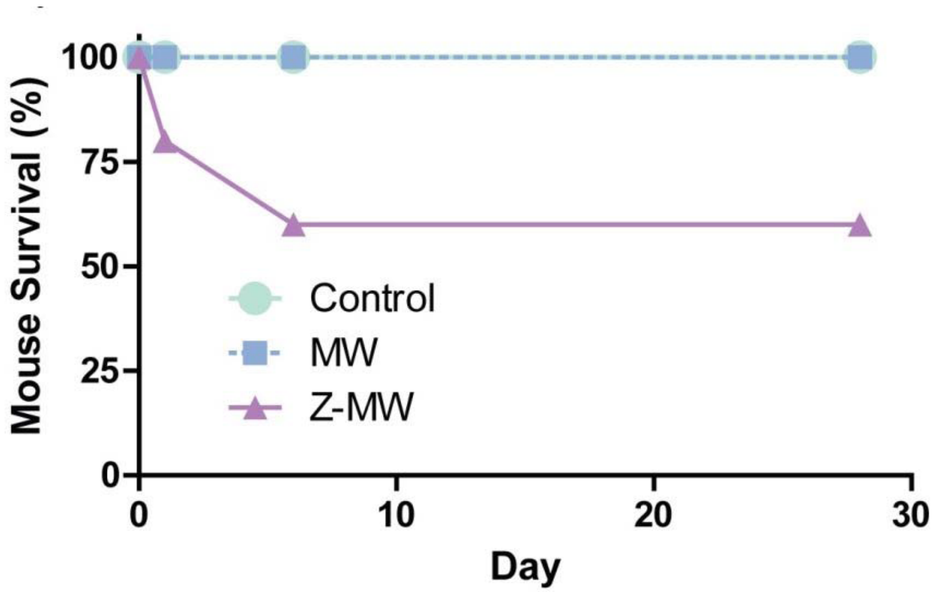

Additional File 6. Survival of mice following oropharyngeal aspiration of Z-MWCNTs (Z-MW) or U-MWCNTs (MW). At one day post-exposure mice treated with Z-MW were lethargic and exhibited shivering, while mice exposed to MW were asymptomatic. N=5 per group at time 0.
